# Immunotherapy in Advanced Prostate Cancer: Current Knowledge and Future Directions

**DOI:** 10.3390/biomedicines10030537

**Published:** 2022-02-24

**Authors:** Fernando López-Campos, Pablo Gajate, Nuria Romero-Laorden, Juan Zafra-Martín, Manel Juan, Susana Hernando Polo, Antonio Conde Moreno, Felipe Couñago

**Affiliations:** 1 Radiation Oncology Department, Hospital Universitario Ramón y Cajal, 28024 Madrid, Spain; 2 Medical Oncology Department, Hospital Universitario Ramón y Cajal, 28024 Madrid, Spain; pgajate@oncologiahrc.com; 3 Medical Oncology Department, Hospital Universitario La Princesa, 28006 Madrid, Spain; nuriaromerolaorden@gmail.com; 4 Department of Radiation Oncology, Hospital Universitario Virgen de la Victoria, 29010 Malaga, Spain; jzafra08@gmail.com; 5 Servei d’Immunologia, CDB-Hospital Clínic, Plataforma de Inmunoterapia HSJD-Clínic, 08036 Barcelona, Spain; mjuan@clinic.cat; 6 Medical Oncology Department, Hospital Universitario Fundación Alcorcón, 28922 Alcorcón, Spain; shernando@salud.madrid.org; 7 Radiation Oncology Department, Hospital Universitario y Politécnico La Fe, 46026 Valencia, Spain; antoniojconde@gmail.com; 8 Department of Radiation Oncology, Hospital Universitario Quirónsalud, 28223 Madrid, Spain; fcounago@gmail.com; 9 Department of Radiation Oncology, Hospital La Luz, 28003 Madrid, Spain; 10 Universidad Europea de Madrid, 28670 Madrid, Spain

**Keywords:** immunotherapy, metastatic castration-resistant prostate cancer, advanced prostate cancer, cancer vaccines, immune checkpoints inhibitors

## Abstract

The advent of immunotherapy has revolutionized cancer treatment. Unfortunately, this has not been the case for metastatic castration-resistant prostate cancer (mCRPC), likely due to the heterogeneous and immune-suppressive microenvironment present in prostate cancer. The identification of molecular biomarkers that could predict response to immunotherapy represents one of the current challenges in this clinical scenario. The management of advanced castration-resistant prostate cancer is rapidly evolving and immunotherapy treatments, mostly consisting of immune checkpoint inhibitors combinations, BiTE^®^ (bispecific T-cell engager) immune therapies, and chimeric antigen receptors (CAR) are in development with promising results. This review analyses the current evidence of immunotherapy treatments for mCRPC, evaluating past failures and promising approaches and discussing the directions for future research.

## 1. Overview of Prostate Cancer Immunology

Prostate cancer is the most common genitourinary tumor in men worldwide and is associated with a significant epidemiological burden, with more than 1.4 million cases worldwide and more than 375.000 associated deaths [1]. Immunotherapy has shifted the treatment paradigm of various genitourinary tumors and is now considered standard in several clinical scenarios [2,3]. However, it has not shown a clear impact in prostate cancer [4].

Exome sequencing of patients with prostate cancer has revealed a low tumor mutational burden (TMB) even in heavily pre-treated castration-resistant prostate cancer (CRPC) patients [5]. This fact contrasts with other tumors such as melanoma, which are more sensitive to immune checkpoint inhibitors [6]. This low TMB may explain (at least partially) the low immunogenicity of prostate cancer [7,8]. Prostate cancer is viewed as a “cold” tumor with components that are predominantly immunosuppressive, such as transforming growth factor ß (TGFß) and regulatory T cells (Tregs). Although tumor cells express a great deal of specific antigens, such as prostate-specific antigen (PSA) and prostate-specific membrane antigen (PSMA), prostate cancer has a low Immunoscore [9]. An imbalance in the immune system can favor this situation of immunotolerance to the tumor; this phenomenon depends on the balance between four factors [10]:

1. Imbalance of cytotoxic cells and Tregs in favor of the latter. Prostate cancer has a low number of tumor-infiltrating lymphocytes, although with a predominance of CD4+ Tregs and M2 macrophages as opposed to CD8+ T lymphocytes and natural killer (NK) cells.
2. Exhaustion of cytotoxic and antigen-presenting dendritic cells due to the overexpression of antigens that block the immune response, such as programmed-cell death protein 1 (PD-1) and cytotoxic T lymphocyte antigen 4 (CTLA-4). This is known as an exhausted phenotype. Despite this high expression of PD-1 and CTLA-4 (though not of programmed-cell death ligand 1 or PD-L1), the response to immune checkpoint inhibitors in these types of tumors is poor.
3. Preponderance of suppressive cytokines released by CD4+ T lymphocytes and M2 macrophages. These cytokines, in addition to inhibiting the immune response, favor tumoral angiogenesis, metastasis and castration resistance. The most relevant suppressive cytokines present in prostate cancer are interleukin (IL) 10, IL-23, TGFß, and certain compounds such as chemokine (C-C motif) ligand (CCL) 2 and CCL22.
4. Intratumoral molecules such as decoy receptor 3 (Dcr3), a soluble receptor and member of the superfamily of tumor necrosis factor receptor (TNFR) that favors tumor growth through TNFR inhibition.

In terms of the natural history of the disease, changes in the tumor microenvironment can be present in the different stages of prostate cancer. Recent studies have shown variations in the tumor microenvironment of disseminated prostate cancer versus localized stages, the first being immunologically “colder” [9]. Bone metastases, the most common site of distant disease, present low intratumoral lymphocyte infiltration, with a predominance of Th17 in contrast to Th1 and high levels of TGFß and IL6. These changes condition the lack of activation of CD8 lymphocytes and NK cells, resulting in a situation of tumor immunotolerance and low response to immunotherapy [11].

**Figure 1:**
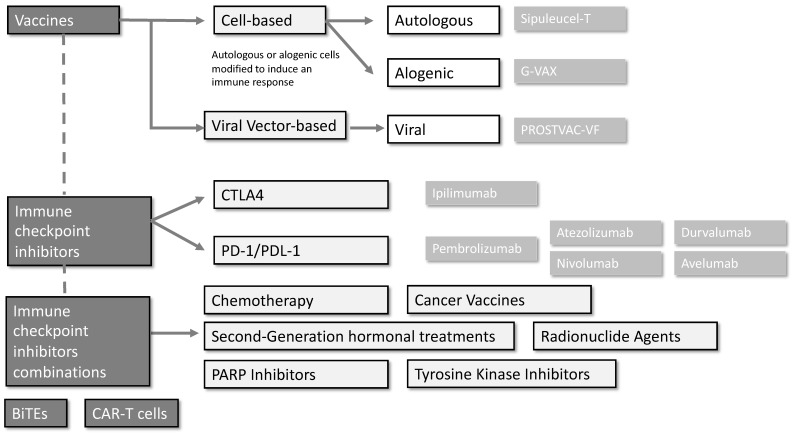
Immunotherapy treatment strategies in metastatic castration-resistant prostate cancer.

At present, there are several immunotherapies that try to take advantage of the biological and molecular characteristics of this disease with the aim of optimizing the available treatment strategies (Figure 1). The identification of biomarkers to predict response to immunotherapy in advanced prostate cancer is currently key to guiding future clinical developments. In this narrative review, we analyze the available evidence for immunotherapy treatments in advanced prostate cancer, as well as ongoing clinical trials and future directions in the treatment of this disease. 

## 2. Vaccines

The treatment with immunomodulatory drugs for prostate cancer includes both passive approaches, such as the direct administration of monoclonal antibodies with high specificity for tumor-associated antigens (TAAs), and active methods such as vaccines, designed with the objective of stimulating an adaptative immune response through antigen presentation [4] Prostate cancer is adequate for analyzing the efficacy of anticancer vaccines due to its biological characteristics, which include a slow growth, early diagnosis of recurrences, and a range of well-known TAAs such as PSA, PSMA, prostate acid phosphatase (PAP), prostate stem cell antigen (PSCA), prostate cancer antigen 3 (PCA3), mucin-1, and six-transmembrane epithelial antigens of the prostate (STEAP) [12]. In addition, vaccines can be used safely in combination with other standard therapies including docetaxel, second-generation hormonal treatments, or radiotherapy [13,14].

However, an effective immune response to a specific TAA might be variable, limited by human leukocyte antigen (HLA) expression and haplotype, which affects the presentation of the immunogenic epitopes [15,16]. Thus, the identification and selection of high-affinity peptide major histocompatibility complex (MHC) may be a predictive factor to consider in this therapeutic strategy to increase the effectiveness of vaccine-induced immunogenicity [17,18].

Vaccine therapies can be grouped in four large groups based on their origin: peptide-based, deoxyribonucleic acid (DNA)-based, cell-based and viral vector-based vaccines [19]. These last two have had the most clinical development yet.

### 2.1. Cell-Based Vaccines

These are formed by autologous or allogenic cells modified to induce an immune response.

#### 2.1.1. Sipuleucel-T (Provenge®)

This is a vaccine of autologous dendritic cells that unleashes an immune response against PAP antigen. The preparation of the vaccine includes the leukapheresis of peripheral mononuclear cells of the patient, ex vivo exposure to a fusion protein that contains PAP antigen and granulocyte-macrophage colony-stimulating factor (GM-CSF) for 36–44 h and the subsequent infusion in the patient for a total of three cycles, each cycle separated by two weeks [20,21].

At present, it is the only anticancer vaccine approved by the Food and Drug Administration (FDA) in patients with asymptomatic or minimally symptomatic mCRPC with no visceral metastases. This approval was granted following the results of the D9901 and D9902A trials [22,23] and later in the phase III trial IMPACT, which reported an improvement in median overall survival (OS) versus placebo: 25.8 vs. 21.7 months (HR 0.78, CI 0.61–0.98; *p* = 0.03), even with a 50% crossover between groups [20,24]. In contrast, no significant differences in progression-free survival (PFS) have been observed (14.6 months for Sipuleucel-T vs. 14.4 for placebo) [20,24], and the Agency for Healthcare Research and Quality has pointed out some limitations in these studies that might have influenced results, such as differences in the subsequent treatments that these patients received [23,25,26].

Recently, the PROCEED study has confirmed its initially published data with the report of additional security and tolerability data for this treatment. A highlight is an ample PFS between the end of Sipuleucel-T and the following treatment that was administered in these patients, which would translate into a significant clinical benefit [26]. Moreover, preliminary security and efficacy data from combinations of Sipuleucel-T with atezolizumab or Radium-223 in mCRPR are already available, although still in development phases [27,28].

#### 2.1.2. G-VAX

G-VAX is based on irradiated tumor cells that have been genetically modified to express GM-CSF with the aim of favoring the growth and differentiation of dendritic cells [29]. Prostate cancer cells are extracted from two cell lines: one hormone-sensitive (LNCaP) and one hormone-resistant (PC3) [30]. This strategy has the advantage of inducing responses to multiple TAAs without the need for HLA pairing [31]. Although results were initially promising in asymptomatic mCRPC, subsequent results from the phase III trials VITAL 1 and VITAL 2 in patients with asymptomatic mCRPC and symptomatic mCRPC, respectively, were negative in terms of OS against docetaxel.

### 2.2. Viral Vector-Based Vaccines

Vector-based vaccines can include vectors derived from oncolytic viruses on the principle that these can infect tumor cells and induce their self-destruction, or bacterial vectors that are actively phagocytized by antigen-presenting cells and, therefore, can generate TAAs and allow for T cell responses [19].

#### PROSTVAC-VF

Poxvirus recombinant vaccine that contains a PSA transgene with an HLA-A2 epitope that has been modified to improve the immunogenicity and a triad of costimulatory molecules: lymphocyte function-associated antigen-3 (LFA-3 or CD58), B7-1 (CD80) and Intercellular Adhesion Molecule 1 (ICAM-1 or CD54) [32]. This vector induces a strong immune response both against PSA and the viral protein, which leads to the destruction of the tumor [32,33,34].

From a clinical standpoint, the published results of PROSTVAC-VF in monotherapy have not demonstrated a clear clinical benefit in patients with mCRPC [35]. Despite a phase II randomized study suggesting an improvement in OS of 25.1 months vs. 16.6 months (HR 0.56, CI 0.37–0.85; *p* = 0.006), the phase III trial PROSPECT, this one designed with OS as the primary endpoint, did not find significant differences and was prematurely closed [35]. However, concomitant PROSVAC-VF and docetaxel has been analyzed in mCRPC with favorable PFS data compared to chemotherapy (CT) alone [13].

Numerous studies are currently researching the administration of PROSTVAC-VF together with immune checkpoint inhibitors such as nivolumab in patients with localized prostate cancer and CRPC (NCT02933255) and nivolumab/ipilimumab in hormone sensitive prostate cancer (NCT03532217). Furthermore, combinations with other immunomodulatory agents are being tested, such as the bifunctional fusion protein bMSB011359C, directed against PD-L1 and TGFß, in patients with recurrence after definitive treatment of the primary tumor [15,19,32,36,37].

## 3. Single-Agent Immune Checkpoint Inhibitors

Immune checkpoint inhibitors are monoclonal antibodies that target different receptors located in key steps of the immune response [38]. The most clinically developed are those directed against PD-1, PD-L1 and CTLA4 [39], with their use being widely accepted in several neoplastic diseases [40,41]. However, the developments in prostate cancer have been more modest.

### 3.1. CTLA-4 Inhibitors

CTLA-4 is a receptor located in the cell membrane of T lymphocytes. Its stimulation conduces to the inhibition of T lymphocyte function [42]. Ipilimumab is an anti-CTLA-4 that has showed favorable, although discreet, results for prostate cancer in several early studies [43,44]. The most recent, by Subudhi et al., included 30 patients with mCRPC and reported an OS of 24.3 months with a median follow-up of 45.5 months [7].

In contrast, we have data on asymptomatic or minimally symptomatic mCRPC with bone metastases and pre-docetaxel from a phase III trial (CA184-095) showing no significant differences in OS: 28.7 vs. 29.7 months (HR 1.11, CI 0.88–1.39; *p* = 0.37) between ipilimumab (10 mg/kg every 3 weeks for up to four doses) and placebo. Moreover, grade 4–5 toxicity was observed in 27% of patients in the ipilimumab group vs. 2% in the placebo arm [45]. A second phase III study (CA 184–043) evaluated ipilimumab against placebo in patients with mCRPC, post-docetaxel, adding an 8 Gy dose of radiotherapy to the bone. This trial was also negative, with an OS of 11.2 months with ipilimumab vs. 10 months with placebo (HR 0.85, CI 0.72–1.00; *p* = 0.05). Pre-specified analysis of this study, however, did show benefit in certain subgroups, particularly in those with a favorable prognosis [46]. Updated results with an additional 2.4 years of follow-up have reported a three, four, and five-year OS between two and three times higher in the ipilimumab arm [47]. These findings suggest that the antitumor effect of ipilimumab could be relevant long-term.

### 3.2. PD-1 and PD-L1 Inhibitors

PD-1 is a T cell transmembrane protein that interacts with its ligand, PD-L1, that is expressed by tumor cells [48].

At present, the use of anti-PD-1/PD-L1 in mCRPC is restricted to clinical trials, given the current lack of evidence to support their utility in clinical practice. Phase I studies with nivolumab (anti-PD-1) and avelumab (anti-PD-L1) that included 17 and 18 patients with mCRPC, respectively [49,50], did not show any objective responses. However, in the last study, 7 patients maintained stable disease after 24 months of treatment [50]. The anti-PD-1 pembrolizumab has been assessed as a monotherapy in multiple clinical trials for prostate cancer. In the phase I trial KEYNOTE-028, pembrolizumab achieved response rates of 17.4% in patients with mCRPC with measurable disease and positive PD-L1 expression [51]. Subsequently, it was evaluated in cohorts 1, 2, and 3 of the phase II study KEYNOTE-199, which included patients with mCRPC previously treated with docetaxel and one or more lines of second-generation hormonal therapy (cohort 1: PD-L1 positive, defined as a combined positivity score [CPS] ≥ 1; cohort 2: PD-L1 negative; cohort 3: non-measurable disease, regardless of PD-L1 status). With a median follow-up of 16.8 months, median OS was 9.5, 7.9, and 14.1 months for cohorts 1, 2, and 3, respectively. The objective response rate (ORR) was poor, with 5% in cohort 1 and 3% in cohort 2. No significant differences were observed between cohorts, although patients with BRCA1/2 or ATM mutations had an ORR of 11% vs. 3% in those without defects in these genes [52].

### 3.3. Biomarkers

Despite the low overall response to immune checkpoint inhibitors, there is a subset of patients who achieve a sustained response to immune checkpoint inhibitors in advanced prostate cancer. In patients treated with ipilumumab, it has been reported how anti-CTLA-4 can instigate T cell responses to tumor neoantigens regardless of TMB. These patients seem to have a greater intratumoral cluster of CD8 T cells, high interferon gamma (IFNγ) response gene signature, and/or antigen-specific T cell responses [7]. On the other hand, an increase of myeloid-derived suppressor cells (MDSCs) in the tumor microenvironment has been associated with resistance to treatment and poor prognosis [53,54]. Additionally, it has been reported how conventional treatments can affect the density of tumor-infiltrating T cells. All these factors should be considered when designing strategies that involve immunotherapy in pretreated patients [55].

High PD-1/PD-L1 expression has been reported in approximately 20% of prostate cancer tumors, but its association with response to immune checkpoint inhibitors remains controversial [56]. It is important to highlight the limitations regarding PD-L1 analysis that may cause bias. Thus, there is neither consensus on the use of these antibodies, nor on the analysis methods applied in different trials. Moreover, levels of PD-L1 expression detected at the primary tumor can vary after treatment and cancer evolution. A decrease in PD-L1 positivity has been reported in series of patients following treatment with neoadjuvant abiraterone plus prednisone before radical prostatectomy [55].

Although still under investigation, other possible biomarkers of response to immunotherapy are DNA repair defects. A phase 2 trial in enriched AR-V7 population has shown higher rates of response with anti-PD1/anti-CTLA4 in DNA repair defects positive, pre-treated CRPC patients (ORR 40%) [57]. Poly-ADP-ribose polymerase (PARP) inhibitors have multiple synergistic effects when combined with immune checkpoint inhibitors, such as increased intratumoral CD8 T cell infiltration, increased IFNγ production and PD-L1 upregulation, as reported in preclinical models [58,59] Trials combining anti-PD-1 and PARP inhibitors are currently ongoing, and preliminary results have shown activity in a selected DNA repair defects positive population [60].

Inactivating mutations in cyclin-dependent kinase 12 (CDK12) have been associated with an increased sensitivity to immunotherapy. CDK12 intervenes in the repair of the association of DNA replication and the biallelic inactivation of CDK12 results in a unique genomic signature, characterized by focal tandem duplications that lead to increased gene fusions and marked differential gene expression [61]. However, there is a low level of clinical evidence to support its use [62]. Further clinical trials are currently ongoing.

Up to date, only the presence of mismatch repair-deficiency (dMMR) has been accepted by clinical guidelines as a transversal agnostic indication for anti-PD1 monotherapy in patients who have progressed to at least one line of previous systemic treatment [63]. Pembrolizumab received approval to treat dMMR patients who do not have an appropriate alternative treatment [64]. However, the percentage of prostate cancer patients with dMMR is low, estimated at around 3% [65], and the evidence for its use is limited. Abida et al. published a series of 11 patients treated with PD-1/PD-L1 therapy that reported 54% of biochemical and 36% of radiological responses, highlighting the existence of durable responders [66]. Given that not all patients with dMMR phenotype respond, further studies should explore resistance mechanisms.

## 4. Immune Checkpoint Inhibitor Combinations

Given that a considerable number of patients are not going to respond to immune checkpoint inhibitors, studies in the last few years are investigating alternative strategies. A particularly active field is the combination of immune checkpoint inhibitors with second-generation hormonal therapies, CT, or PARP inhibitors (Table 1).

### 4.1. Anti-CTLA-4 and anti-PD-1/PD-L1 Combinations

The combination of anti-CTLA-4 and anti-D-1/PD-L1 has showed a synergistic effect with excellent results in melanoma and renal cancer [67,68]. It has been observed that patients with androgen receptor splice variant 7 (AR-V7) expression present a greater number of alterations in DNA repair genes that would make them more susceptible to immune checkpoint inhibitors [69]. In this context, the combination of nivolumab and ipilimumab was first evaluated in 15 patients with mCRPC and AR-V7 expression. However, the combination was effective exclusively in those patients with AR-V7 expression and alterations in DNA repair genes, with differences in PSA response rate (PSA RR) (33% vs. 0%; *p* = 0.14), ORR (40% vs. 0%; *p* = 0.46), radiographic progression-free survival (rPFS) (HR 0.31; *p* = 0.01), and OS (HR 0.41; *p* = 0.11).

The phase II non-randomized study CheckMate-650 also evaluated this combination in 90 patients with mCRPC divided in two cohorts: pre-CT (45 patients) and post-CT (45 patients) [70]. An ORR of 25% and 10%, and OS of 19 and 15.2 months, respectively, was reported. However, four treatment-related deaths occurred, and 42–53% of patients presented grade 3–4 adverse effects. In terms of biomarkers, ORR was 36.4% vs. 12.1, in favor of patients with PD-L1 expression (in the 63 patients evaluable for this endpoint).

A phase II study randomized 52 patients with mCRPC in progression after abiraterone or enzalutamide to receive durvalumab or durvalumab plus ipilimumab [71]. In the combination arm 16% of patients responded, whereas none of the patients in the monotherapy arm did.

### 4.2. Immune Checkpoint Inhibitors and Chemotherapy

CT has an immunomodulatory effect on the tumor by regulating the composition and immunosuppressive pathways of the tumor microenvironment, favoring the release of antigens, and stimulating the activity of cytotoxic T lymphocytes [72,73,74,75]. For these reasons, the combination of CT and immune checkpoint inhibitors is being investigated for mCRPC.

Cohort B of the phase II trial CheckMate 9KD evaluated the combination of docetaxel and nivolumab in 41 patients with mCRPC in progression after second-generation hormonal therapy and CT-naïve. Results of an interim analysis were presented at ESMO 2019. ORR was 36.8% in patients with measurable disease, PSA RR was 46.3%, and rPFS was 8.2 months [76]. Cohort B of the phase Ib/II KEYNOTE-365 trial analyzed the combination of docetaxel and pembrolizumab in 104 patients of the same characteristics. ORR was 18%, PSA RR was 28%, rPFS was 8.3 months, and OS was 20.4 months [77].

After these results, two phase III trials, CheckMate7DX and Keynote-921 are evaluating the combination of docetaxel with nivolumab and pembrolizumab, respectively. These studies will confirm if the combination is superior to CT alone in these patients.

### 4.3. Immune Checkpoint Inhibitors and Second-Generation Hormonal Treatments

The resistance to enzalutamide is associated with an increased expression of PD-L1 in dendritic cells [78]. However, the immunomodulatory role of the new hormonal therapies is controversial [79,80].

The IMbassador 250 study compared enzalutamide plus atezolizumab against enzalutamide plus placebo y patients with mCRPC in progression to abiraterone and docetaxel. After the inclusion of 759, the study was closed prematurely due to the absence of impact in OS (15.2 months vs. 16.6 months; HR 1.12, CI 95% 0.91–1.37; *p* = 0.28). No differences were observed in ORR, PSA RR, or rPFS [81].

The KEYNOTE-199 trial evaluated the combination of enzalutamide and pembrolizumab in two cohorts of patients with mCRPC refractory to enzalutamide (cohort 4: measurable disease; cohort 5: predominantly bone disease). In cohort 4 12% of patients had a response, with a disease control rate (DCR) of 51%. In cohort 5, DCR was also 51%. rPFS was four months in both cohorts [52]. In cohort C of the phase Ib/II KEYNOTE-365 study, patients with mCRPC that had progressed to abiraterone received enzalutamide plus pembrolizumab. They included 103 patients with a PSA RR of 22%, an ORR of 12% in patients with measurable disease, and a DCR of 32% [82]. In addition, there is a phase III trial ongoing that will compare treatment with enzalutamide plus pembrolizumab/placebo in CT-naïve mCRPC patients (NCT03834493), and a cohort of the CheckMate 9KD is also studying the combination of nivolumab and enzalutamide in this subset (NCT03338790).

### 4.4. Immune Checkpoint Inhibitors and PARP Inhibitors

PARP inhibitors can have immunomodulatory effects on various levels. The microsatellite instability associated with alterations in DNA repair genes can act as a predictive biomarker of response to immunotherapy [83]. Preclinical models have shown that treatment with PARP inhibitors can produce an overexpression of PD-L1 [59]. Moreover, it has been reported that olaparib induces an increase in the sensitivity of NK cells in prostate cancer [84].

In cohort A of the KEYNOTE-365 study, patients with mCRPC that have progressed to docetaxel and second-generation hormonal therapies received treatment with olaparib plus pembrolizumab. Eighty-four patients were included, with a PSA RR of 9%, ORR of 8.3%, rPFS of 4 months and OS of 14 months [85]. The phase III trial KEYLYNK-010 is also evaluating this combination in this group of patients (NCT03834519). Cohort A of CheckMate 9KD is currently studying the combination of nivolumab and rucaparib in mCRPC before and after CT. Preliminary results of the patients who had not received previous CT were recently presented at ESMO 2021. PSA RR was 27.3%, ORR was 15.4, rPFS was 8.1 months, and OS was 20.2 months [86]. However, the response in patients without a deficit in homologous recombination was very limited.

A phase II study has evaluated the combination of durvalumab and olaparib in 17 patients with mCRPC after progression to abiraterone and/or enzalutamide. rPFS was 16.1 months and 53% had a serological or radiographic response. Patients with alterations in DNA repair genes had a rPFS of 16.1 months and an ORR of 83% [60].

### 4.5. Immune Checkpoint Inhibitors and Cancer Vaccines

A phase Ib study has evaluated the combination of Sipuleucel T and atezolizumab in two arms of sequential treatment: atezolizumab followed by Sipuleucel T and Sipuleucel followed by atezolizumab. Thirty-seven patients were included, with an ORR after 6 months of 8% and a DCR of 41%. rPFS was 8.2 months in arm 1 vs. 5.8 months in arm 2 [27].

In addition, a phase II trial has studied the effectiveness of Sipuleucel T in combination with ipilimumab, reporting a PSA RR of 10% and rPFS of 5.72 months [87].

### 4.6. Immune Checkpoint Inhibitors with Tyrosine Kinase Inhibitors

There is an interaction between the angiogenesis pathway and the immune response that favors the generation of an immunosuppressive state in the tumor microenvironment. In this context, the treatment with antiangiogenics has immunomodulatory effects that can facilitate the response to immune checkpoint inhibitors [88].

The phase Ib study COSMIC 021 evaluated the combination of cabozantinib and atezolizumab in mCRPC. Recently, the results of the expansion phase of patients previously treated with abiraterone or enzalutamide were presented at ESMO 2021. With 132 patients included, ORR was 15% and DCR was 81%. rPFS was 5.7 months and OS was 18.4 months. This effect was also consistent in subgroups with worse prognosis such as those with visceral disease [89].

### 4.7. Immune Checkpoint Inhibitors with Radionuclide Agents

A phase Ib trial has evaluated the combination of atezolizumab and Radium-223 in mCRPC. However, the clinical response was low, with an ORR of 6.8%, PSA RR of 4.5%, and rPFS of 3 months [90]. An alternative combination of Radium-223 with pembrolizumab is being tested in phase II trial (NCT03093428).

Recently, ^177^Lu-PSMA-617 has reported effectiveness in mCRPC [91]. Combinations of this treatment with other drugs, such as immune checkpoint inhibitors, are currently being tested. At ESMO 2021, results of the phase Ib/II PRINCE trial have been presented. Thirty-seven patients with mCRPC in progression to a line of second-generation hormonal therapy or docetaxel were treated with ^177^Lu-PSMA-617 and pembrolizumab. PSA RR was 73%, ORR was 78% in patients with measurable disease, and rPFS was 65% [92].

## 5. Future Directions

Once the importance of immunotherapy for the treatment of mCRPC has been defined by clearly establishing its effectiveness beyond immune checkpoint inhibitors, the option of cell therapy has reappeared, specifically with the use of adoptive cell therapies (ACT) with T lymphocytes.

In this regard, one of the most revolutionary proposals has come from the genetic engineering of T cells [93]. After reporting its efficacy in hematological tumors, therapy with chimeric antigenic receptors (CAR) in T cells has become the preferred option for new research on solid tumors, especially mCRPC [94], due to the expression of TAAs in these tumors. Following the achievements of molecules that target PSMA such as ^177^Lu-PSMA-617, this surface molecule has become the main candidate to test CAR-T therapy in prostate cancer, and several research proposals have appeared in recent years. Although the importance of tumor infiltrating T lymphocytes was established years ago [95], their direct use as adoptive cell therapy in prostate cancer has not been developed as much as in other tumors. It has been only in the last few years [96] that other immunotherapies have regained interest, not just against PSMA but also anti-PSA, anti-PAP, anti-PSCA, or other more general tumor associated antigens such as B7-H3 [97] or Epithelial Cell Adhesion Molecule (EpCAM) precursor [94].

In any case, it is the anti-PSMA CAR-T therapy which is currently ahead in clinical trials (NCT01140373, NCT00664196, NCT03089203) [98]. Although very serious adverse effects have been described in some of its patients, especially when TGFβ is simultaneously blocked, the “race” to have an approved CAR-PSMA continues, and verifiable results are expected to arrive next year (Table 2).

**Figure 2:**
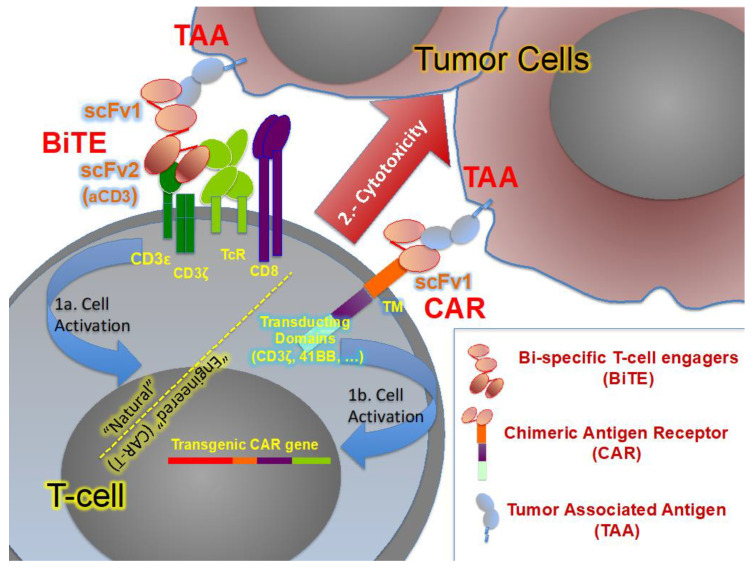
Comparison between the mechanisms of BiTE and CAR in T cells to activate (1) these lymphocytes after the recognition of the corresponding tumor associated antigen TAA. After activation, these T cells exert an antitumoral effect (2), mainly through cytotoxicity.

Another interesting strategy is the use of bi-specific T cell engagers (BiTEs). These are antibodies generated from the variable chains of two different monoclonal antibodies. An anti-CD3 domain is found in all BiTEs, whereas the other antibody binds to an antigen such as PSMA. The transitory union between CD3 and this TAA unleashes the activation of T cells (Figure 2), (Table 3).

On the other hand, a promising strategy based on TCR isolation for neoepitope-specific TCR cloning and engineering of autologous CD8+ and CD4+ T-cells is being implemented in clinical trials that include patients with prostate cancer (NCT03970382).

There is a clear consensus among specialists in the field that the future for immunotherapy will come through simultaneous or sequential combinations of several targets and options, including cell immunotherapy. It is not unreasonable to think that improving the cellular response with initial vaccination with dendritic cells, followed by the sustained use of immune checkpoint inhibitors and culminating with genetically modified T cells, could be one of these future combinations. To arrive at this point, however, there is still much work to be done regarding single-agent immunotherapy to guarantee the safety and affordability of each of these strategies.

## 6. Conclusions

Immunotherapy is still being evaluated for the treatment of advanced prostate cancer through different strategies that will hopefully define its definitive place among the available therapeutic options in this disease. The results of monotherapy with anti-CTLA-4, anti-PD-1, and anti-PD-L1 have been modest, and only pembrolizumab in those patients with dMMR has an established clinical use in the context of tumor-agnostic therapy. Up to date, no biomarkers have been identified that may allow us to select those patients that will benefit the most from these treatments. Over the last few years, we have seen some promising preliminary results from combinations of not only conventional therapies with immunomodulatory drugs, but also dual immunotherapy and alternative strategies such as targeted anti-PSMA immunotherapy that make us hopeful for future results of ongoing studies.

## Figures and Tables

**Table 1:** Selected clinical trials of ICI in combination with prostate cancer therapies.

Treatment	Clinical Phase	Eligibility	Sample Size	Current Stage	Trial Identification
**ICI combinations**					
Nivolumab + Ipilimumab	II	mCRPC expressing AR-V7	15	Active, not recruiting	NCT02601014
Nivolumab + Ipilimumab	II	mCRPC Cohort 1 (pre-chemotherapy), cohort 2 (post-chemotherapy)	90	Active, recruiting	NCT02985957
Durvalumab +/− Tremelimumab	II	mCRPC after prior NHT, and no more than one taxane	52	Active, not recruiting	NCT02788773
**ICI + chemotherapy**					
Nivolumab + Docetaxel	II	Chemotherapy naïve mCRPC after prior NHT	41	Active, not recruiting	NCT03338790
Nivolumab + Docetaxel	III	Chemotherapy naïve mCRPC after prior NHT	984	Active, recruiting	NCT04100018
Pembrolizumab + Docetaxel	Ib/II	Chemotherapy naïve mCRPC after prior NHT	104	Active, recruiting	NCT02861573
Pembrolizumab + Docetaxel	III	Chemotherapy naïve mCRPC after prior NHT	1000	Active, recruiting	NCT03834506
**ICI + Novel hormonal therapies**					
Pembrolizumab + Enzalutamide	II	Chemotherapy naïve mCRPC after prior enzalutamide	126	Active, not recruiting	NCT02787005
Pembrolizumab + Enzalutamide	Ib/II	Chemotherapy naïve mCRPC after prior abiraterone	103	Active, recruiting	NCT02861573
Pembrolizumab + Enzalutamide	III	Chemotherapy naïve mCRPC	1200	Active, recruiting	NCT03834493
Atezolizumab + Enzalutamide	III	mCRPC after prior abiraterone and docetaxel	759	Active, not recruiting	NCT03016312
Nivolumab + Enzalutamida	II	mCRPC	330	Active, not recruiting	NCT03338790
**ICI + PARP inhibitors**					
Pembrolizumab + Olaparib	Ib/II	mCRPC after prior docetaxel and ≤2 NHT	84	Active, recruiting	NCT02861573
Pembrolizumab + Olaparib	III	mCRPC after prior docetaxel and 1 NHT	780	Active, not recruiting	NCT03834519
Nivolumab + Rucaparib	II	mCRPC Cohort 1 (pre-chemotherapy), cohort 2 (post-chemotherapy)	71 (Cohort 1)	Active, not recruiting	NCT03338790
Durvalumab + Olaparib	II	mCRPC after prior NHT	17	Completed	NCT02484404
**ICI + vaccines**					
Atezolizumab + Sipuleucel-T	Ib	Asymptomatic or minimally symptomatic progressive mCRPC	37	Completed	NCT03024216
Ipilimumab + Sipuleucel-T	II	mCRPC	50	Completed	NCT01804465
Nivolumab + PROSTVAC	I/II	mCRPC	29	Active, recruiting	NCT02933255
Nivolumab + ChAdOx1-MVA 5T4	II	mCRPC	23	Active, not recruiting	NCT03815942
**ICI + tyrosine kinase inhibitors**					
Atezolizumab + Cabozantinib	Ib	mCRPC after 1 prior NHT	132	Active, recruiting	NCT03170960
**ICI + radionuclide**					
Atezolizumab + Radium 223	Ib	mCRPC	44	Completed	NCT02814669
Pembrolizumab + Radium 223	II	mCRPC	45	Active, not recruiting	NCT03093428
Nivolumab + Radium 223	II	mCRPC	36	Active, recruiting	NCT04109729
Pembrolizumab + ^177Lu^-PSMA	Ib	mCRPC after prior abiraterone and docetaxel	37	Active, not recruiting	NCT03658447

**Table 2:** Ongoing clinical trials with CAR-T therapy in metastatic castration-resistant prostate cancer. CARm: CAR macrophages; CHMC: City of Hope Medical Center; EpCAM: epithelial cell adhesion molecule; KLK2: Kallikrein 2; NCI: National Cancer Institute; PSCA: prostate stem cell antigen; PSMA: prostate-specific membrane antigen; TGFβRdn: dominant negative TGFβ receptor; TMpPSMA: PSMA Target Module.

NCT Number	Title	CAR	Location	Sponsors
NCT04768608	PD1 Integrated Anti-PSMA CART in Treating Patients with Castrate-Resist Prostate Cancer.	PD1 integrat -PSMA CART	Hangzhou, Zhejiang, China (1)	Zhejiang University
	PSCA-CAR T Cells in Treating Patients With PSCA+ Metastatic Castration Resistant Prostate Cancer	PSCA CART	Duarte, CA, United States (1)	CHMC/NCI
NCT04227275	A Study of CART-PSMA-TGFβRDN in Patients With Metastatic Castration Resistant Prostate Cancer	PSMA-TGFβRdn CART	United States (9)	Tmunity Therapeutics
NCT04249947	P-PSMA-101 CAR-T Cells in the Treatment of Subjects With Metastatic Castration-Resistant Prostate Cancer (mCRPC)	pPSMA CART	United States (7)	Poseida Therapeutics, Inc.
NCT05022849	A Study of JNJ-75229414 for Metastatic Castration-resistant Prostate Cancer Participants	KLK2 CART	United States (7)	Janssen Research & Development
NCT02744287	Safety and Activity Study of PSCA-Targeted CAR-T Cells (BPX-601) in Subjects With Selected Advanced Solid Tumors	PSCA CART	United States (7)	Bellicum Pharmaceuticals
NCT03013712	Clinical Research of CAR T Cells Targeting EpCAM Positive Cancer (CARTEPC)	EpCAM CART	Chendu, China (1)	1st Affiliated Hos Chengdu Med College
NCT04107142	Haplo/Allogen NKG2DL-targeting Chimeric Antigen Receptor-grafted γδ T Cells for Relapsed or Refractory Solid Tumor	NKG2DL CART	Malaysia (1)	CytoMed Therapeutics Pte Ltd.
NCT04660929	CAR-macrophages for the Treatment of HER2 Overexpressing Solid Tumors	HER2 CARm	United States (3)	Carisma Therap Inc
NCT04633148	Dose-escalating trial with UniCAR02-T Cells and PSMA Target Module (TMpPSMA) in patients with Progressive Disease After Standard Systemic Therapy in Cancers with Positive PSMA Marker	UniCAR02-T pPSMA	Germany (4)	Cellex Patient Treatment/PHARMALOG
NCT04429451	PSMA-specific CAR-T Cell Therapy	PSMA CART	Shenzhen, Guangdong, China (4)	Shenzhen Geno-Imm

**Table 3:** Ongoing clinical trials with BITE therapy in metastatic castration-resistant prostate cancer. HLE: half-life extended; PSMA: prostate-specific membrane antigen; STEAP1: six transmembrane epithelial antigen of the prostate 1.

NCT Number	Title	BiTE	Location (n. of Centers)	Sponsors
NCT04631601	Safety and Efficacy of Therapy for Metastatic Castration-resistant Prostate Cancer (mCRPC)	Acapatamab (HLE anti-PSMA-CD3)	USA, Canada Europe, Australia (13)	Amgen
NCT03792841	Safety, Tolerability, Pharmacokinetics, and Efficacy of Acapatamab in Subjects With mCRPC	Acapatamab (HLE anti-PSMA-CD3)	USA, Europe, Australia, Japan, Singapore (27)	Amgen
NCT01723475	First-in-man Dose Escalation Study of BAY2010112 in … Prostate Cancer	MT110 (anti-PSMA-CD3)	Austria, Germany (5)	Bayer
NCT00635596	Phase I Study of MT110 in Lung … Prostate and Ovarian Cancer (MT110-101)	MT110 (anti-PSMA-CD3)	Germany (4)	Amgen
NCT04221542	Study of AMG 509 in Subjects With Metastatic Castration-Resistant Prostate Cancer	AMG 509 (anti-STEAP1-CD3)	USA, Canada, East Asia, Australia (17)	Amgen

## Data Availability

Not applicable.

## References

[1] Ferlay J., Colombet M., Soerjomataram I., Parkin D.M., Piñeros M., Znaor A., Bray F. (2021). Cancer statistics for the year 2020: An overview. Int. J. Cancer.

[2] Rhea L.P., Mendez-Marti S., Kim D., Aragon-Ching J.B. (2021). Role of immunotherapy in bladder cancer. Cancer Treat. Res. Commun..

[3] Braun D.A., Bakouny Z., Hirsch L., Flippot R., Van Allen E.M., Wu C.J., Choueiri T.K. (2021). Beyond conventional immune-checkpoint inhibition—Novel immunotherapies for renal cell carcinoma. Nat. Rev. Clin. Oncol..

[4] Bansal D., Reimers M.A., Knoche E.M., Pachynski R.K. (2021). Immunotherapy and immunotherapy combinations in metastatic castration-resistant prostate cancer. Cancers.

[5] Grasso C.S., Wu Y.M., Robinson D.R., Cao X., Dhanasekaran S.M., Khan A.P., Quist M.J., Jing X., Lonigro R.J., Brenner C. (2012). The mutational landscape of lethal castration-resistant prostate cancer. Nature.

[6] Davar D., Lin Y., Kirkwood J.M. (2015). Unfolding the mutational landscape of human melanoma. J. Investig. Dermatol..

[7] Subudhi S.K., Vence L., Zhao H., Blando J., Yadav S.S., Xiong Q., Reuben A., Aparicio A., Corn P.G., Chapin B.F. (2020). Neoantigen responses, immune correlates, and favorable outcomes after ipilimumab treatment of patients with prostate cancer. Sci. Transl. Med..

[8] Maleki Vareki S. (2018). High and low mutational burden tumors versus immunologically hot and cold tumors and response to immune checkpoint inhibitors. J. Immunother. Cancer.

[9] Galon J., Bruni D. (2019). Approaches to treat immune hot, altered and cold tumours with combination immunotherapies. Nat. Rev. Drug Discov..

[10] Sun B.L. (2021). Immunotherapy in treatment of metastatic prostate cancer: An approach to circumvent immunosuppressive tumor microenvironment. Prostate.

[11] Jiao S., Subudhi S.K., Aparicio A., Ge Z., Guan B., Miura Y., Sharma P. (2019). Differences in Tumor Microenvironment Dictate T Helper Lineage Polarization and Response to Immune Checkpoint Therapy. Cell.

[12] Prokhnevska N., Emerson D.A., Kissick H.T., Redmond W.L. (2019). Immunological Complexity of the Prostate Cancer Microenvironment Influences the Response to Immunotherapy. Adv. Exp. Med. Biol..

[13] Arlen P.M., Gulley J.L., Parker C., Skarupa L., Pazdur M., Panicali D., Beetham P., Tsang K.Y., Grosenbach D.W., Feldman J. (2006). A randomized phase II study of concurrent docetaxel plus vaccine versus vaccine alone in metastatic androgen-independent prostate cancer. Clin. Cancer Res..

[14] Uhlman M.A., Bing M.T., Lubaroff D.M. (2014). Prostate cancer vaccines in combination with additional treatment modalities. Immunol. Res..

[15] Comber J.D., Philip R. (2014). MHC class I antigen presentation and implications for developing a new generation of therapeutic vaccines. Ther. Adv. Vaccines.

[16] Khalili S., Rahbar M.R., Dezfulian M.H., Jahangiri A. (2015). In silico analyses of Wilms’ tumor protein to designing a novel multi-epitope DNA vaccine against cancer. J. Theor. Biol..

[17] Yu Z., Theoret M.R., Touloukian C.E., Surman D.R., Garman S.C., Feigenbaum L., Baxter T.K., Baker B.M., Restifo N.P. (2004). Poor immunogenicity of a self/tumor antigen derives from peptide–MHC-I instability and is independent of tolerance. J. Clin. Investig..

[18] Engels B., Engelhard V.H., Sidney J., Sette A., Binder D.C., Liu R.B., Kranz D.M., Meredith S.C., Rowley D.A., Schreiber H. (2013). Relapse or eradication of cancer is predicted by peptide-major histocompatibility complex affinity. Cancer Cell.

[19] Adamaki M., Zoumpourlis V. (2021). Immunotherapy as a precision medicine tool for the treatment of prostate cancer. Cancers.

[20] Kantoff P.W., Higano C.S., Shore N.D., Berger E.R., Small E.J., Penson D.F., Redfern C.H., Ferrari A.C., Dreicer R., Sims R.B. (2010). Sipuleucel-T Immunotherapy for Castration-Resistant Prostate Cancer. N. Engl. J. Med..

[21] Drake C.G. (2010). Prostate cancer as a model for tumour immunotherapy. Nat. Rev. Immunol..

[22] Small E.J., Schellhammer P.F., Higano C.S., Redfern C.H., Nemunaitis J.J., Valone F.H., Verjee S.S., Jones L.A., Hershberg R.M. (2006). Placebo-controlled phase III trial of immunologic therapy with Sipuleucel-T (APC8015) in patients with metastatic, asymptomatic hormone refractory prostate cancer. J. Clin. Oncol..

[23] Higano C.S., Schellhammer P.F., Small E.J., Burch P.A., Nemunaitis J., Yuh L., Provost N., Frohlich M.W. (2009). Integrated data from 2 randomized, double-blind, placebo-controlled, phase 3 trials of active cellular immunotherapy with sipuleucel-T in advanced prostate cancer. Cancer.

[24] Beer T.M., Bernstein G.T., Corman J.M., Glode L.M., Hall S.J., Poll W.L., Schellhammer P.F., Jones L.A., Xu Y., Kylstra J.W. (2011). Randomized trial of autologous cellular immunotherapy with sipuleucel-T in androgen-dependent prostate cancer. Clin. Cancer Res..

[25] Mark D., Samson D.J., Bonnell C.J., Ziegler K.M., Aronson N. (2011). Outcomes of Sipuleucel-T Therapy.

[26] Higano C.S., Armstrong A.J., Sartor A.O., Vogelzang N.J., Kantoff P.W., McLeod D.G., Pieczonka C.M., Penson D.F., Shore N.D., Vacirca J. (2019). Real-world outcomes of sipuleucel-T treatment in PROCEED, a prospective registry of men with metastatic castration-resistant prostate cancer. Cancer.

[27] Rosser C.J., Hirasawa Y., Acoba J.D., Tamura D.J., Pal S.K., Huang J., Scholz M.C., Dorffet T.B. (2020). Phase Ib study assessing different sequencing regimens of atezolizumab (anti-PD-L1) and sipuleucel-T (SipT)in patients who have asymptomatic or minimally symptomatic metastatic castrate resistant prostate cancer. J. Clin. Oncol..

[28] Marshall C.H., Fu W., Wang H., Park J.C., DeWeese T.L., Tran P.T., Song D.Y., King S., Afful M., Hurrelbrink J. (2021). Randomized phase II trial of sipuleucel-T with or without radium-223 in men with bone-metastatic castration-resistant prostate cancer. Clin. Cancer Res..

[29] Warren T.L., Weiner G.J. (2000). Uses of granulocyte-macrophage colony-stimulating factor in vaccine development. Curr. Opin. Hematol..

[30] Simons J.W., Carducci M.A., Mikhak B., Lim M., Biedrzycki B., Borellini F., Clift S.M., Hege K.M., Ando D.G., Piantadosi S. (2006). Phase I/II trial of an allogeneic cellular immunotherapy in hormone-naïve prostate cancer. Clin. Cancer Res..

[31] Simmons A.D., Li B., Gonzalez-Edick M., Lin C., Moskalenko M., Du T., Creson J., VanRoey M.J., Jooss K. (2007). GM-CSF-secreting cancer immunotherapies: Preclinical analysis of the mechanism of action. Cancer Immunol. Immunother..

[32] Handa S., Hans B., Goel S., Bashorun H.O., Dovey Z., Tewari A. (2020). Immunotherapy in prostate cancer: Current state and future perspectives. Ther. Adv. Urol..

[33] Gulley J.L., Madan R.A., Tsang K.Y., Jochems C., Marté J.L., Farsaci B., Tucker J.A., Hodge J.W., Liewehr D.J., Steinberg S.M. (2014). Immune impact induced by PROSTVAC (PSA-TRICOM), a therapeutic vaccine for prostate cancer. Cancer Immunol. Res..

[34] Madan R.A., Arlen P.M., Mohebtash M., Hodge J.W., Gulley J.L. (2009). Prostvac-VF: A vector-based vaccine targeting PSA in prostate cancer. Expert Opin. Investig. Drugs.

[35] Gulley J.L., Borre M., Vogelzang N.J., Siobhan N., Agarwal N., Parker C.C., Pook D.W., Rathenborg P., Flaig T.W., Carles J. (2019). Phase III Trial of PROSTVAC in asymptomatic or minimally symptomatic metastatic castration-resistant prostate cancer. J. Clin. Oncol..

[36] Picardo S.L., Hansen A.R. (2019). The PD-1/PD-L1 pathway in advanced prostate cancer—Have we milked this cow?. Ann. Transl. Med..

[37] Strauss J., Heery C.R., Schlom J., Madan R.A., Cao L., Kang Z., Lamping E., Marté J.L., Donahue R.N., Grenga I. (2018). Phase I trial of M7824 (MSB0011359C), a bifunctional fusion protein targeting PD-L1 and TGFb, in advanced solid tumors. Clin. Cancer Res..

[38] Granier C., De Guillebon E., Blanc C., Roussel H., Badoual C., Colin E., Saldmann A., Gey A., Oudard S., Tartour E. (2017). Mechanisms of action and rationale for the use of checkpoint inhibitors in cancer. ESMO Open.

[39] Darvin P., Toor S.M., Sasidharan Nair V., Elkord E. (2018). Immune checkpoint inhibitors: Recent progress and potential biomarkers. Exp. Mol. Med..

[40] Mahoney K.M., Freeman G.J., McDermott D.F. (2015). The next immune-checkpoint inhibitors: Pd-1/pd-l1 blockade in melanoma. Clin. Ther..

[41] Bylicki O., Barazzutti H., Paleiron N., Margery J., Assié J.B., Chouaïd C. (2019). First-Line Treatment of Non-Small-Cell Lung Cancer (NSCLC) with Immune Checkpoint Inhibitors. BioDrugs.

[42] Chen L. (2004). Co-inhibitory molecules of the B7-CD28 family in the control of T-cell immunity. Nat. Rev. Immunol..

[43] Finkelstein S.E., Salenius S., Mantz C.A., Shore N.D., Fernandez E.B., Shulman J., Myslicki F.A., Agassi A.M., Rotterman Y., DeVries T. (2015). Combining immunotherapy and radiation for prostate cancer. Clin. Genitourin Cancer.

[44] Slovin S.F., Higano C.S., Hamid O., Tejwani S., Harzstark A., Alumkal J.J., Scher H.I., Chin K., Gagnier P., McHenry M.B. (2013). Ipilimumab alone or in combination with radiotherapy in metastatic castration-resistant prostate cancer: Results from an open-label, multicenter phase i/ii study. Ann. Oncol..

[45] Beer T.M., Kwon E.D., Drake C.G., Fizazi K., Logothetis C., Gravis G., Ganju V., Polikoff J., Saad F., Humanski P. (2017). Randomized, double-blind, phase III trial of ipilimumab versus placebo in asymptomatic or minimally symptomatic patients with metastatic chemotherapy-naive castration-resistant prostate cancer. J. Clin. Oncol..

[46] Kwon E.D., Drake C.G., Scher H.I., Fizazi K., Bossi A., Van den Eertwegh A.J.M., Krainer M., Houede N., Santos R., Mahammedi H. (2014). Ipilimumab versus placebo after radiotherapy in patients with metastatic castration-resistant prostate cancer that had progressed after docetaxel chemotherapy (CA184-043): A multicentre, randomised, double-blind, phase 3 trial. Lancet Oncol..

[47] Fizazi K., Drake C.G., Beer T.M., Kwon E.D., Scher H.I., Gerritsen W.R., Bossi A., van den Eertwegh A.J.M., Krainer M., Houede N. (2020). Final Analysis of the Ipilimumab Versus Placebo Following Radiotherapy Phase III Trial in Postdocetaxel Metastatic Castration-resistant Prostate Cancer Identifies an Excess of Long-term Survivors. Eur. Urol..

[48] Francisco L.M., Salinas V.H., Brown K.E., Vanguri V.K., Freeman G.J., Kuchroo V.K. (2009). PD-L1 regulates the development, maintenance, and function of induced regulatory T cells. J. Exp. Med..

[49] Topalian S.L., Hodi F.S., Brahmer J.R., Gettinger S.N., Smith D.C., McDermott D.F., Powderly J.D., Carvajal R.D., Sosman J.A., Atkins M.B. (2012). Safety, Activity, and Immune Correlates of Anti–PD-1 Antibody in Cancer. N. Engl. J. Med..

[50] Fakhrejahani F., Madan R.A., Dahut W.L., Karzai F., Cordes L.M., Schlom J., Liow E., Bennett C., Zheng T., Yu J. (2017). Avelumab in metastatic castration-resistant prostate cancer (mCRPC). J. Clin. Oncol..

[51] Hansen A.R., Massard C., Ott P.A., Haas N.B., Lopez J.S., Ejadi S., Wallmark J.M., Keam B., Delord J.-P., Aggarwal R. (2018). Pembrolizumab for advanced prostate adenocarcinoma: Findings of the KEYNOTE-028 study. Ann. Oncol..

[52] Hoimes C.J., Graff J.N., Tagawa S.T., Hwang C., Kilari D., Ten Tije A.J., Omlin A.U., McDermott R.S., Vaishampayan U.N., Tony Elliott T. (2020). KEYNOTE-199 cohorts (C) 4 and 5: Phase II study of pembrolizumab (pembro) plus enzalutamide (enza) for enza-resistant metastatic castration-resistant prostate cancer (mCRPC). J. Clin. Oncol..

[53] Tesi R.J. (2019). MDSC; the Most Important Cell You Have Never Heard of. Trends Pharmacol. Sci..

[54] Zhang S., Ma X., Zhu C., Liu L., Wang G., Yuan X. (2016). The role of myeloid-derived suppressor cells in patients with solid tumors: A meta-analysis. PLoS ONE.

[55] Calagua C., Russo J., Sun Y., Schaefer R., Lis R., Zhang Z., Mahoney K., Bubley G.J., Loda M., Taplin M.E. (2017). Expression of PD-L1 in hormone-naïve and treated prostate cancer patients receiving neoadjuvant abiraterone acetate plus prednisone and leuprolide. Clin. Cancer Res..

[56] Massari F., Ciccarese C., Caliò A., Munari E., Cima L., Porcaro A.B., Novella G., Artibani W., Sava T., Eccher A. (2016). Magnitude of PD-1, PD-L1 and T Lymphocyte Expression on Tissue from Castration-Resistant Prostate Adenocarcinoma: An Exploratory Analysis. Target. Oncol..

[57] Boudadi K., Suzman D.L., Anagnostou V., Fu W., Luber B., Wang H., Niknafs N., White J.R., Silberstein J.L., Sullivan R. (2018). Ipilimumab plus nivolumab and DNA-repair defects in AR-V7-expressing metastatic prostate cancer. Oncotarget.

[58] Huang J., Wang L., Cong Z., Amoozgar Z., Kiner E., Xing D., Orsulic S., Matulonis U., Goldberg M.S. (2015). The PARP1 inhibitor BMN 673 exhibits immunoregulatory effects in a Brca1-/- murine model of ovarian cancer. Biochem. Biophys. Res. Commun..

[59] Jiao S., Xia W., Yamaguchi H., Wei Y., Chen M.K., Hsu J.M., Hsu J.L., Yu W.H., Du Y., Lee H.H. (2017). PARP inhibitor upregulates PD-L1 expression and enhances cancer-associated immunosuppression. Clin. Cancer Res..

[60] Karzai F., Vanderweele D., Madan R.A., Owens H., Cordes L.M., Hankin A., Couvillon A., Nichols E., Bilusic M., Beshiri M.L. (2018). Activity of durvalumab plus olaparib in metastatic castration-resistant prostate cancer in men with and without DNA damage repair mutations 11 Medical and Health Sciences 1112 Oncology and Carcinogenesis. J. Immunother. Cancer.

[61] Wu Y.M., Cieślik M., Lonigro R.J., Vats P., Reimers M.A., Cao X., Ning Y., Wang L., Kunju L.P., de Sarkar N. (2018). Inactivation of CDK12 Delineates a Distinct Immunogenic Class of Advanced Prostate Cancer. Cell.

[62] Antonarakis E.S., Isaacsson Velho P., Fu W., Wang H., Agarwal N., Santos V.S., Maughan B.L., Pili R., Adra N., Sternberg C.N. (2020). CDK12 -Altered Prostate Cancer: Clinical Features and Therapeutic Outcomes to Standard Systemic Therapies, Poly (ADP-Ribose) Polymerase Inhibitors, and PD-1 Inhibitors. JCO Precis. Oncol..

[63] Schaeffer E., Srinivas S., Antonarakis E.S., Armstrong A.J., Bekelman J.E., Cheng H., D’Amico A.V., Davis B.J., Desai N., Dorff T. (2021). Prostate cancer, version 1.2021: Featured updates to the nccn guidelines. JNCCN J. Natl. Compr. Cancer Netw..

[64] Le D.T., Uram J.N., Wang H., Bartlett B.R., Kemberling H., Eyring A.D., Skora A.D., Luber B.S., Azad N.S., Laheru D. (2015). PD-1 Blockade in Tumors with Mismatch-Repair Deficiency. N. Engl. J. Med..

[65] Robinson D., Van Allen E.M., Wu Y.M., Schultz N., Lonigro R.J., Mosquera J.M., Montgomery B., Taplin M.E., Pritchard C.C., Attard G. (2015). Integrative clinical genomics of advanced prostate cancer. Cell.

[66] Abida W., Cheng M.L., Armenia J., Middha S., Autio K.A., Vargas H.A., Rathkopf D., Morris M.J., Danila D.C., Slovin S.F. (2019). Analysis of the Prevalence of Microsatellite Instability in Prostate Cancer and Response to Immune Checkpoint Blockade. JAMA Oncol..

[67] Wolchok J.D., Chiarion-Sileni V., Gonzalez R., Rutkowski P., Grob J.-J., Cowey C.L., Lao C.D., Wagstaff J., Schadendorf D., Ferrucci P.F. (2017). Overall Survival with Combined Nivolumab and Ipilimumab in Advanced Melanoma. N. Engl. J. Med..

[68] Motzer R.J., Tannir N.M., McDermott D.F., Arén Frontera O., Melichar B., Choueiri T.K., Plimack E.R., Barthélémy P., Porta C., George S. (2018). Nivolumab plus Ipilimumab versus Sunitinib in Advanced Renal-Cell Carcinoma. N. Engl. J. Med..

[69] Joshi H., Pinski J.K. (2016). Association of ARV7 expression with molecular and clinical characteristics in prostate cancer. J. Clin. Oncol..

[70] Sharma P., Pachynski R.K., Narayan V., Fléchon A., Gravis G., Galsky M.D., Mahammedi H., Patnaik A., Subudhi K.S., Ciprotti M. (2020). Nivolumab Plus Ipilimumab for Metastatic Castration-Resistant Prostate Cancer: Preliminary Analysis of Patients in the CheckMate 650 Trial. Cancer Cell.

[71] Hotte S.J., Winquist E., Chi K.N., Ellard S.L., Sridhar S., Emmenegger U., Jindal S., Tidwell R.S.S., Varma A., Logothetis C.J. (2019). CCTG IND 232: A phase II study of durvalumab with or without tremelimumab in patients with metastatic castration resistant prostate cancer (mCRPC). Ann. Oncol..

[72] Hodge J.W., Garnett C.T., Farsaci B., Palena C., Tsang K.Y., Ferrone S., Gameiro S.R. (2013). Chemotherapy-induced immunogenic modulation of tumor cells enhances killing by cytotoxic T lymphocytes and is distinct from immunogenic cell death. Int. J. Cancer.

[73] Galluzzi L., Buqué A., Kepp O., Zitvogel L., Kroemer G. (2015). Immunological Effects of Conventional Chemotherapy and Targeted Anticancer Agents. Cancer Cell.

[74] Emens L.A., Middleton G. (2015). The interplay of immunotherapy and chemotherapy: Harnessing potential synergies. Cancer Immunol. Res..

[75] Chan O.T.M., Yang L.X. (2000). The immunological effects of taxanes. Cancer Immunol. Immunother..

[76] Fizazi K., Gonzalez Mella P., Castellano D., Minatta J.N., Rezazadeh Kalebasty A., Shaffer D., Vazquez-Limon J.C., Armstrong A.J., Sanchez-Lopez H.M., Sharkey B. (2019). Efficacy and safety of nivolumab in combination with docetaxel in men with metastatic castration-resistant prostate cancer in CheckMate 9KD. Ann. Oncol..

[77] Sridhar S.S., Kolinsky M.P., Gravis G., Mourey L., Piulats Rodriguez J.M.M., Romano E., Berry W.R., Gurney H., Retz M., Appleman L.J. (2020). Pembrolizumab (pembro) plus docetaxel and prednisone in patients (pts) with abiraterone acetate (abi) or enzalutamide (enza)-pretreated metastatic castration-resistant prostate cancer (mCRPC): KEYNOTE-365 cohort B efficacy, safety and, biomarker results. J. Clin. Oncol..

[78] Bishop J.L., Sio A., Angeles A., Roberts M.E., Azad A.A., Chi K.N., Zoubeidi A. (2015). PD-L1 is highly expressed in Enzalutamide resistant prostate cancer. Oncotarget.

[79] Ardiani A., Gameiro S.R., Kwilas A.R., Donahue R.N., Hodge J.W. (2014). Androgen deprivation therapy sensitizes prostate cancer cells to T-cell killing through androgen receptor dependent modulation of the apoptotic pathway. Oncotarget.

[80] Pal S.K., Moreira D., Won H., White S.W., Duttagupta P., Lucia M., Jones J., Hsu J., Kortylewski M. (2019). Reduced T-cell numbers and elevated levels of immunomodulatory cytokines in metastatic prostate cancer patients de novo resistant to abiraterone and/or enzalutamide therapy. Int. J. Mol. Sci..

[81] Sweeney C.J., Gillessen S., Rathkopf D., Matsubara N., Drake C., Fizazi K., Piulats J.M., Wysocki P.J., Buchschacher G.L., Doss J. (2020). Abstract CT014: IMbassador250: A phase III trial comparing atezolizumab with enzalutamide vs enzalutamide alone in patients with metastatic castration-resistant prostate cancer (mCRPC). Cancer Res..

[82] Conter H.J., Shore N.D., Berry W.R., Fong P.C.C., Piulats Rodriguez J.M.M., Appleman L.J., Todenhöfer T., Gravis G., Laguerre B., Gurney H. (2020). Pembrolizumab (pembro) plus enzalutamide (enza) in patients (pts) with abiraterone acetate (abi)-pretreated metastatic castration-resistant prostate cancer (mCRPC): KEYNOTE-365 cohort C efficacy, safety, and biomarker results. J. Clin. Oncol..

[83] Peyraud F., Italiano A. (2020). Combined parp inhibition and immune checkpoint therapy in solid tumors. Cancers.

[84] Fenerty K.E., Padget M., Wolfson B., Gameiro S.R., Su Z., Lee J.H., Rabizadeh S., Soon-Shiong P., Hodge J.W. (2018). Immunotherapy utilizing the combination of natural killer- and antibody dependent cellular cytotoxicity (ADCC)-mediating agents with poly (ADP-ribose) polymerase (PARP) inhibition 11 Medical and Health Sciences 1112 Oncology and Carcinogenesis 11 Medical. J. Immunother. Cancer.

[85] Yu E.Y., Piulats J.M., Gravis G., Laguerre B., Arranz Arija J.A., Oudard S., Fong P.C.C., Kolinsky M.P., Augustin M., Feyerabend S. (2020). KEYNOTE-365 cohort A updated results: Pembrolizumab (pembro) plus olaparib in docetaxel-pretreated patients (pts) with metastatic castration-resistant prostate cancer (mCRPC). J. Clin. Oncol..

[86] Petrylak D.P., Perez-Gracia J.L., Lacombe L., Bastos D.A., Mahammedi H., Kwan E.M., Zschäbitz S., Armstrong A.J., Pachynski R.K., Goh J.C. (2021). 579MO CheckMate 9KD cohort A2 final analysis: Nivolumab (NIVO) + rucaparib for chemotherapy (CT)-naïve metastatic castration-resistant prostate cancer (mCRPC). Ann. Oncol..

[87] Sinha M., Zhang L., Subudhi S., Chen B., Marquez J., Liu E.V., Allaire K., Cheung A., Ng S., Nguyen C. (2021). Pre-existing immune status associated with response to combination of sipuleucel-T and ipilimumab in patients with metastatic castration-resistant prostate cancer. J. Immunother. Cancer.

[88] Tripathi M., Nandana S., Billet S., Cavassani K.A., Mishra R., Chung L.W.K., Posadas E.M., Bhowmick N.A. (2017). Modulation of cabozantinib efficacy by the prostate tumor microenvironment. Oncotarget.

[89] Agarwal N., McGregor B.A., Maughan B.L., Dorff T., Kelly W., Fang B., McKay R., Singh P., Pagliaro L., Dreicer R. (2021). LBA24 Cabozantinib (C) in combination with atezolizumab (A) in patients (pts) with metastatic castration-resistant prostate cancer (mCRPC): Results of expanded cohort 6 of the COSMIC-021 study. Ann. Oncol..

[90] Morris M.J., Fong L., Petrylak D.P., Sartor A.O., Higano C.S., Pagliaro L.C., Alva A.S., Appleman L.J., Tan W., Vaishampayan U.N. (2020). Safety and clinical activity of atezolizumab (atezo) + radium-223 dichloride (r-223) in 2L metastatic castration-resistant prostate cancer (mCRPC): Results from a phase Ib clinical trial. J. Clin. Oncol..

[91] Sartor O., de Bono J., Chi K.N., Fizazi K., Herrmann K., Rahbar K., Tagawa S.T., Nordquist L.T., Vaishampayan N., El-Hadda G. (2021). Lutetium-177-PSMA-617 for Metastatic Castration-Resistant Prostate Cancer. N. Engl. J. Med..

[92] Sandhu S.K., Joshua A.M., Emmett L., Spain L., Horvath L.G., Crumbaker M., Anton A., Wallace R., Pasam A., Bressel M. (2021). 577O PRINCE: Interim analysis of the phase Ib study of 177Lu-PSMA-617 in combination with pembrolizumab for metastatic castration resistant prostate cancer (mCRPC). Ann. Oncol..

[93] Pantuck M., Palaskas N., Drakaki A. (2018). Next generation T-cell therapy for genitourinary malignancies, part B: Overcoming obstacles and future strategies for success. Cancer Treat. Res. Commun..

[94] Schepisi G., Cursano M.C., Casadei C., Menna C., Altavilla A., Lolli C., Cerchione C., Paganelli G., Santini D., Tonini G. (2019). CAR-T cell therapy: A potential new strategy against prostate cancer. J. Immunother. Cancer.

[95] Haas G.P., Solomon D., Rosenberg S.A. (1990). Tumor-infiltrating lymphocytes from nonrenal urological malignancies. Cancer Immunol. Immunother..

[96] Yunger S., Bar El A., Zeltzer L., Fridman E., Raviv G., Laufer M., Schachter J., Markel G., Itzhaki O., Besser M.J. (2019). Tumor-infiltrating lymphocytes from human prostate tumors reveal anti-tumor reactivity and potential for adoptive cell therapy. Oncoimmunology.

[97] Yang S., Wei W., Zhao Q. (2020). B7-H3, a checkpoint molecule, as a target for cancer immunotherapy. Int. J. Biol. Sci..

[98] Yu Y.D., Kim T.J. (2021). Chimeric antigen receptor-engineered T cell therapy for the management of patients with metastatic prostate cancer: A comprehensive review. Int. J. Mol. Sci..

